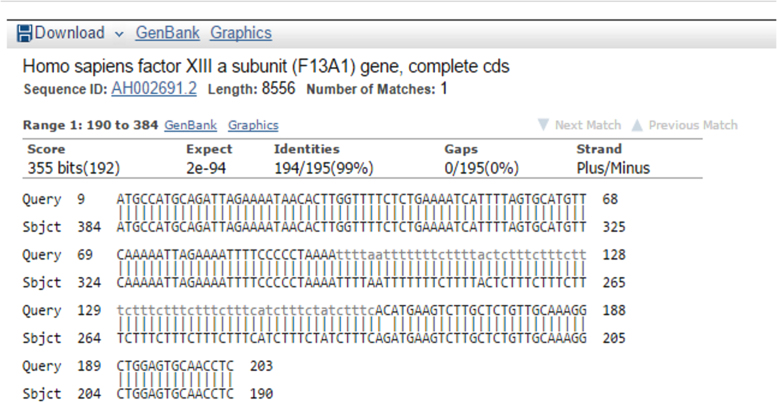# Use of coagulation factor XIII (F13) gene as an internal control for normalization of genomic DNA’s for HLA typing

**DOI:** 10.1016/j.mex.2018.07.020

**Published:** 2018-08-03

**Authors:** Anupama Cheleri Neduvat, Prerana Madhusudhana Murthy, Sudarson Sundarrajan, Sriram Padmanabhan

**Affiliations:** Cancyte Technologies Pvt. Ltd, Rangadore Memorial Hospital, 1st Cross, Shankarapuram, Bangalore 560004, India

**Keywords:** Use of coagulation factor XIII (F13) gene as an internal control for normalization of genomic DNA’s for HLA typing, DNA, Electrophoresis, Extraction, Factor XIII, Human blood, PCR, Spectrophotometry, Spin-column

## Abstract

Genomic DNA (gDNA) obtained from whole blood samples is a critical element for genomic research and clinical diagnosis. PCR efficiencies of the targeted genes like HLA-A, -B, -C, DPB1 and DRB1 using such isolated gDNAs were variable in spite of having similar amounts of gDNA taken for PCR. We addressed such PCR variabilities by normalizing the gDNA’s using an internal control of human coagulation factor XIII that was found to be variable with all samples and did not correlate with the observed A_260_ nm readings. The PCR and Q-PCR methodologies for the human coagulation factor XIII have been optimized, and the advantages of normalizing gDNA preparations based on F13 copy numbers have been discussed. This method will serve as a suitable choice to be used in laboratories and research centres, particularly when dealing with a large number of samples for the next-generation sequencing purposes, and in forensic labs with limited sample availability.

## Graphical abstract

**Figure:**
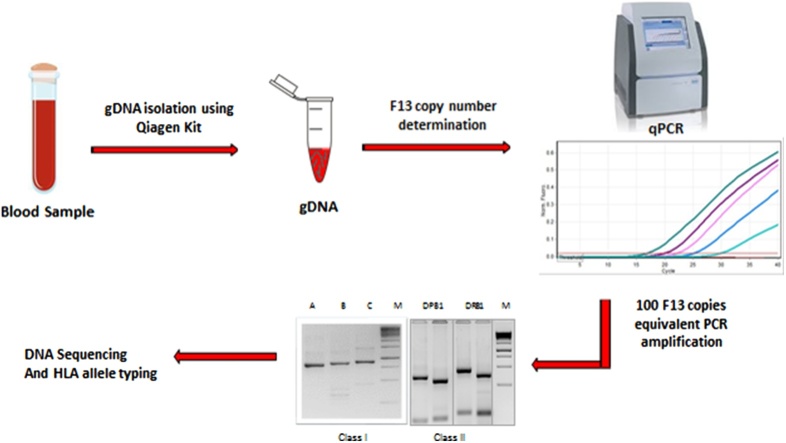

**Specifications table**

**Table:** 

Subject area	Biochemistry, Genetics and Molecular Biology
More specific subject area	Quantitation of genomic DNA
Method name	Use of Coagulation Factor XIII (F13) Gene as an Internal Control for Normalization of Genomic DNA’s for HLA Typing
Name and reference of original method	P. Khare, V. Raj, S. Chandra, S. Agarwal, Quantitative and qualitative assessment of DNA extracted from saliva for its use in forensic identification. J. Forensic Dent. Sci. 6 (2014) 81–85.
Resource availability	Not applicable

## Background

Preparation of pure human genomic DNA (gDNA) from whole blood in appreciable quantities is critical for basic science research, genetics, metagenomics, and clinical diagnosis. Of all the methods available for isolation of human gDNA [1,2], spin column technology appears to be a relatively simple approach to extract nucleic acids from small amounts of biological samples [3,4]. The sensitivity of polymerase chain reaction (PCR) assays is decided by the purity of gDNA employed as templates, and hence, determining the purity of the gDNA preparations for any downstream applications assumes critical importance. It has been reported that there is a steep rise in the average primer-dimer rate and PCR cross-overs with increasing numbers of PCR cycles at DNA concentrations below 30 ng/μl, especially for applications of human leucocyte antigen (HLA) typing [5,6]. Hence, we realized that alternate methods to determine accurate concentrations of gDNAs would be immensely useful for metagenomics studies and other applications. We detailed out experiments carried out on the use of human coagulation factor XIII (F13) gene as an internal control (IC) for accurate quantification of gDNA and demonstration of the usefulness of this approach in HLA typing.

## Method details

### Method name

F13 gene as internal control for genomic DNA quantitation

### Materials

• Isopropyl alcohol (Analytical Grade)
• Commercially available spin column kits (QiaAmp DNA Blood Mini Kit, Qiagen, GmbH).
• Oligonucleotides were synthesized from BioServe Technologies, Hyderabad, India.
• DMSO, BSA, Tween-20 were procured from Sigma Chemical Co., St. Louis, USA.
• Taq DNA polymerase and pTZ57R/T vector (Thermo Fisher Scientific, USA)
• LB-agar plates, LB broth and ampicillin (Hi-Media, Mumbai, India)
• 1X Thermo Scientific Maxima SYBR green master mix (Thermo Fisher Scientific, USA)

### Procedure

#### Collection of blood samples

Written consent forms from all donors at the point of collection for the purposes of developing a Bone Marrow (BM) registry for organ and BM transplantation was obtained by BMCDT-Infosys Bone Marrow Registry, India. These consent forms are in accordance with the regulatory body guidelines. Specific approval from the local ethics committee was not sought as the purpose of the study was to assess the method of HLA typing in comparison with the existing HLA typing techniques. No new genetic information outside of the HLA genes that would affect the donors of the material used in this study has been gained.

#### Genomic DNA isolation

Since Qiagen columns have superior DNA properties with least PCR inhibitors [7], we used these columns for isolation of gDNA following the manufacturer’s protocols. Briefly, 200 μl of whole blood was first processed for a lysis step (RBC lysis), followed by lysis of the nucleated cells and binding of the lysed material to the column (silica membrane). After washing the column to remove the contaminants, the bound gDNA was eluted using appropriate elution buffer as recommended by the manufacturer.

#### HLA PCR

HLA-Class I and Class II antigens have been shown to be highly polymorphic in individuals and are usually the panel used by researchers for HLA typing [8,9]. Human gDNA of Sample A (40 ng/μl) prepared from all the three extraction kits was used as the template to assess the relative efficiency of the preparations on HLA PCR of Class I genes, namely exons 2 and 3 of HLA-A, -B and -C. PCR was carried out in 25 μl reaction volume, and the PCR mixture comprised of 1 X ammonium sulfate buffer, pH 8.3, 2.5 mM magnesium chloride, 0.2 mM dNTP’s, 5% DMSO, 2% Tween-20, 0.32% BSA, and 0.4 μM of HLA primers [9]. The HLA-A PCR was done using HLA-A-Exon 2 forward and HLA-Exon-3 reverse primers, HLA-B PCR used HLA-B-Exon 2 forward and Exon 3 reverse primers, and HLA-C PCR used HLA-C-Exon 2 forward and HLA-C-Exon 3 reverse primers (Table 1). The primers for Exons 2 and 3 of Class II genes DPB1 and DRB1 was designed based on exon regions as per Lange et al. [8]. The PCR condition was as follows: Initial denaturation of 95 °C, 5 min, followed by 28 cycles of denaturation step of 95 °C for 30 s, 60 °C for 30 s, and 72 °C for 45 s. The final extension step was done at 72 °C for 10 min, and suitable aliquots were loaded on 2% agarose gel for visualization.

**Table 1:** Oligonucleotides used in this study.

Primer	Target gene	Primer sequence 5’ to 3’	Reference
Forward F13	*F13A1*	GAGGTTGCACTCCAGCCTTT	Khare et al. [10]
Reverse F13	*F13A1*	ATGCCATGCAGATTAGAAA	Khare et al. [10]
Forward-A-Exon 2	*HLA-A*	AAACGGCCTCTGTGGGGAGAAGCAA	Itoh et al [9]
Reverse A-Exon 3	*HLA-A*	GTGGCCCCTGGTACCCGT	Itoh et al. [9]
Forward B-Exon 2	*HLA-B*	GGGCGGGCAGGAGAGAGGGGACCGCAG	Itoh et al. [9]
Reverse B- Exon 3	*HLA-B*	AGGCCATCCCCGCCGACCTAT	Itoh et al. [9]
Forward-C-Exon 2	*HLA-C*	GAGGTGCCCGCCCGGCGA	Itoh et al. [9]
Reverse-C-Exon 3	*HLA-C*	GCTGATCCCATTTTCCTCCCCTCCTC	Itoh et al. [9]
Forward-DPB1-Exon 2	*DPB1*	GAGGATTAGATGAGAGTGGCGCCT	This study
Reverse-DPB1-Exon 2	*DPB1*	CGGCACTAAGGTCCCTTAGGCCA	This study
Forward-DPB1-Exon 3	*DPB1*	GGAAAGAAGGACAATCTCAAATTC	This study
Reverse-DPB1-Exon 3	*DPB1*	GAGGGTCATCAGAGACTCA	This study
Forward-DRB1-Exon 2	*DRB1*	ATCCTTCGTGTCCCCACAGCA	This study
Reverse-DRB1-Exon 2	*DRB1*	GCTCACCTCGCCGCTGCACTG	This study
Forward-DRB1-Exon 3	*DRB1*	TCCTGACTCATTCCCTCTACCT	This study
Reverse-DRB1-Exon 3	*DRB1*	GAAGTCAGAAAGCTGCTC	This study

#### Construction of pTZ57R/T-F13

The F13 gene was PCR amplified from human gDNA sample using the primer (Table 1) recommended by Khare et al. [10]. The F13A1 gene PCR mix comprised of 40 ng of human gDNA with 1 U of Taq DNA polymerase, 2.5 μl of 10 X ammonium sulfate buffer, pH 8.3 (Thermo Fisher Scientific, USA), 0.2 mM dNTP’s, 2% Tween-20, 0.32% BSA, 5% DMSO and 2.5 mM MgCl_2_ in a final volume of 25 μl master mix. Cycling parameters were 95 °C for 5 min followed by 28 cycles of 94 °C for 30 s, 50 °C for 30 s, and 72 °C for 45 s with a final extension at 72 °C for 10 min A 5 μl aliquot of the PCR reaction was run on a 2% agarose gel stained with ethidium bromide. PCR for F13 gene was carried out by the method described above, unless mentioned otherwise.

The amplified PCR product was purified and ligated into a TA cloning vector pTZ57R/T vector (Thermo Fisher Scientific, USA). The recombinant clones of pTZ57R/T-F13 were subjected to plasmid preparation, followed by DNA sequencing.

#### Gradient PCR for optimal PCR signal for F13 gene

A gradient PCR was set up using equal concentration of pTZ57R/T-F13 plasmid DNA. PCR was initiated with an initial denaturation at 95 °C for 5 min followed by 40 cycles of 95 °C for 30 s and Tm gradient from 50 °C to 65 °C for 30 s and 72 °C for 30 s. The annealing temperatures used for each of the 8 reactions were 50 °C, 51 °C, 52.9 °C, 55.7 °C, 59.1 °C, 62 °C, 63.8 °C, and 65 °C.

#### Optimizing F13 primer concentrations

F13 gene PCR was carried out at 0.4 μM, 0.2 μM, 0.1 μM, 0.04 μM, and 0.004 μM primer concentrations to examine the optimal concentration of primer required to amplify the F13 gene from a known amount of pTZ57R/T-F13 plasmid by Q-PCR with minimum amounts of primer-dimer (PD).

PCR was set up with 1X Thermo Scientific Maxima SYBR green master mix and with 0.5 μl of forward and reverse F13A1 primers (Table 1), each at the above mentioned concentrations and 1 μl of template DNA. The volume was made up to 25 μl with nuclease free water. The Q-PCR was carried out using a Qiagen Q-PCR machine (Qiagen, Germany) with an initial denaturation at 95 °C for 5 min followed by 40 cycles of 95 °C for 30 s, 51.1 °C for 30 s and 72 °C for 30 s, followed by melt-curve analysis to identify the specific amplified product. The combination of primers that yielded optimal assay performance was chosen for further experiments.

#### Limit of detection (LOD) of F13 Q-PCR

Suitable dilutions of pTZ57R/T-F13 plasmid DNA was made in nuclease free water to obtain 10^5^–10^1^ copy numbers in the PCR reaction mix. The PCR was set up in duplicates, with reaction mix containing1X Thermo Scientific Maxima SYBR green master mix and F13 primers (Table 1) and suitable copy numbers of the plasmid. The volume was made up to 25 μl with nuclease free water.

Forty cycles of amplification were performed with the following thermal conditions: Initial denaturation at 95 °C for 5 min followed by 40 cycles of 95 °C for 30 s, 51.1 °C for 30 s and 72 °C for 45 s followed by the melt curve analysis.

#### F13 copy number determination in 10 gDNA donor samples isolated using Qiagen columns and subsequent HLA PCR using 0.2 ng gDNA equivalent to 100 copies of F13

gDNAs obtained from the blood of 10 randomly chosen HLA donor samples were taken, and F13 copy numbers were determined in their respective gDNAs by F13Q-PCR. Subsequently, the F13 copy numbers of all the 10 samples were equalized to 100 F13 copies (equivalent to 0.2 ng gDNA) and then subjected to HLA PCR’s for Class I genes as described earlier. For PCR of exons 2 and 3 of class II genes, DPB1 and DRB1, gDNA’s equivalent to 100 copies of F13 was taken from four randomly selected samples for the PCR.

### Validation of the proposed method

**Fig. 1:**
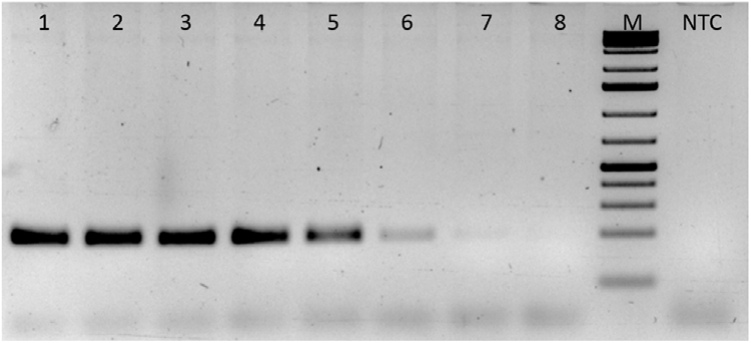
Gradient PCR for F13A1 (F13) gene. Gradient PCR for F13 gene was carried out using specific primers as outlined in the M & M section. Lane 1: 50 °C, lane 2: 51 °C, lane 3:52.9 °C, lane 4: 55.7 °C, lane 5: 59.1 °C, lane 6: 62 °C, lane 7 63.8 °C, lane 8: 65 °C. The F13 PCR showed maximum signal between 50–52.9 °C.M: DNA molecular weight maker (75 bp–10 Kb).

**Fig. 2:**
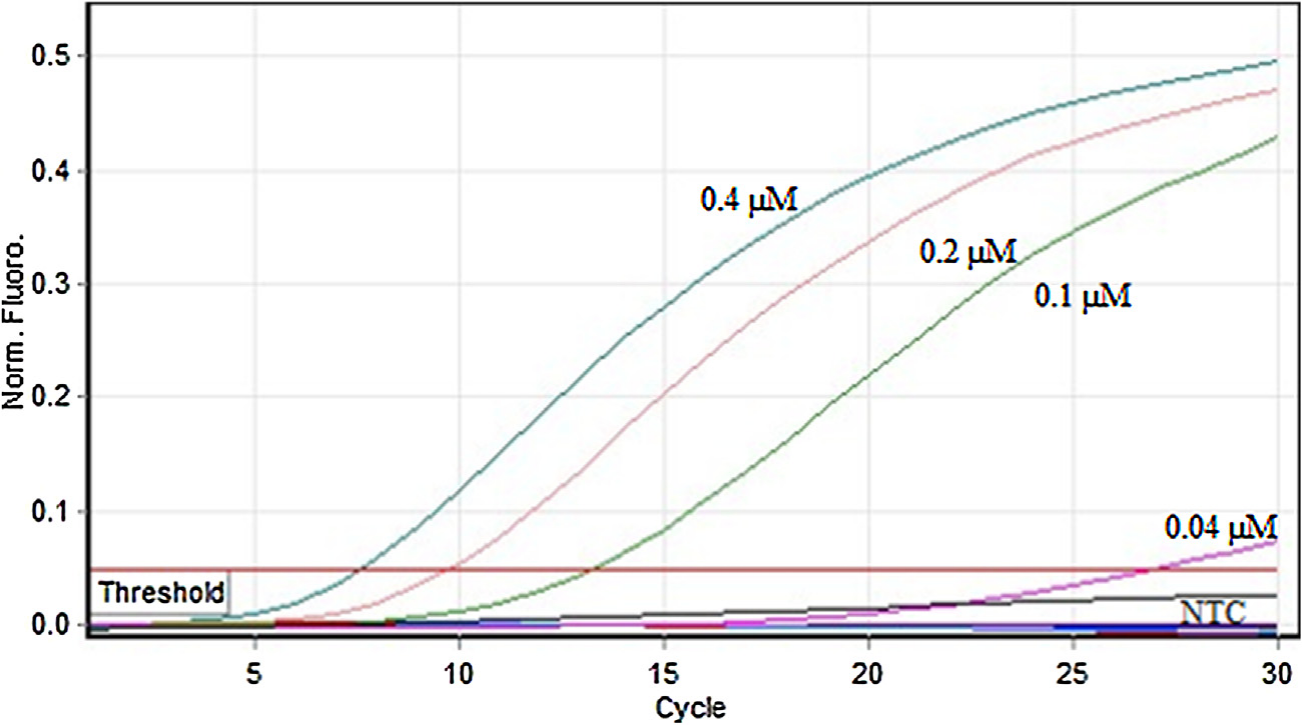
Amplification plot of F13 Q-PCR with different primer concentrations. The F13 PCR was found to be maximal with 0.4 μM primer concentration with no visible primer dimer. Note the absence of any signal in no template control (NTC).

**Fig. 3:**
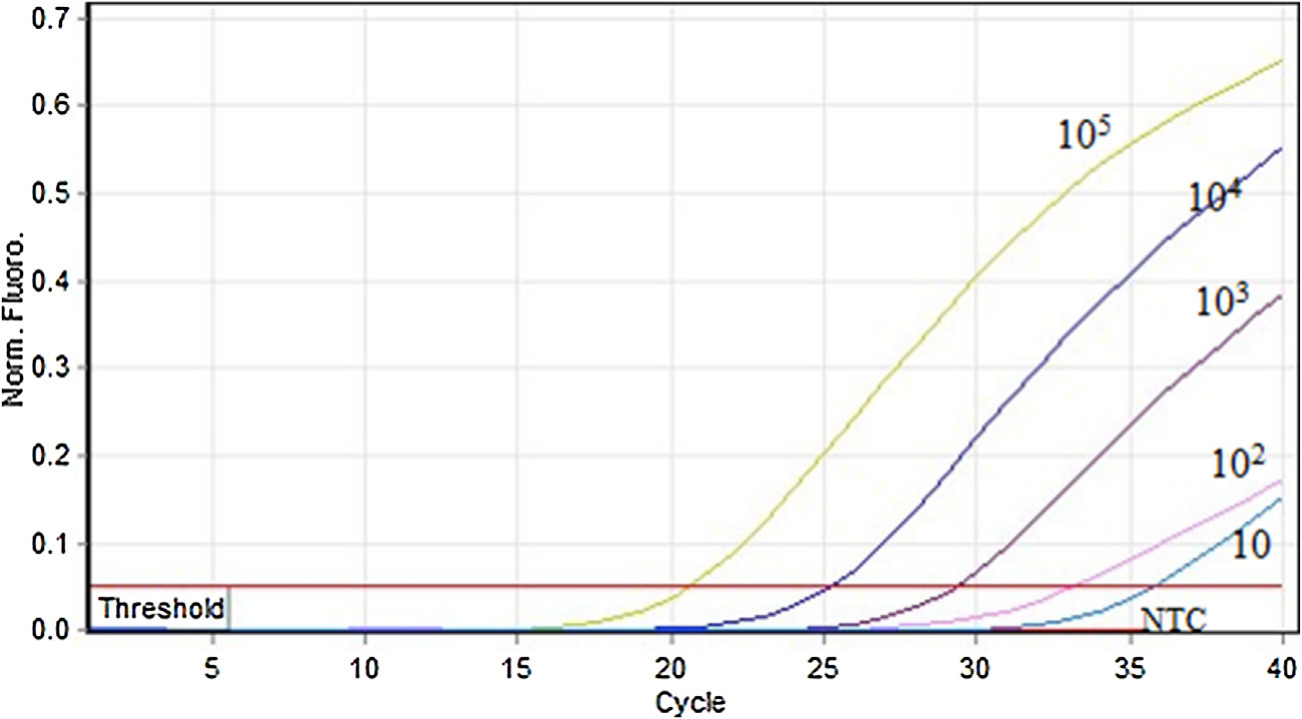
Amplification plot of F13 Q-PCR at different template concentrations. It is evident from the amplification plot that the Ct values changed as the template concentration of F13 were altered. The no template control (NTC) did not show any signal, as expected and the LOD seems to be 10 attg.

The DNA sequence of one of the representative clone of pTZ57R/T-F13 is given in supplementary Fig. S1. It is evident from the DNA sequence that the amplified PCR amplicon belonged to human F13 A1 gene when analyzed and compared against the human F13A1 gene sequence available in the NCBI database. It was observed that the F13 specific primers amplified F13 gene at Tm ranging from 50 °C–59.1 °C, and the optimum activity was seen at 51 °C. (Fig. 1). F13 primers at 0.4 μM final concentration could amplify the F13 product with a Ct of 7.7 (Fig. 2) with no PD. The LOD of the F13 Q-PCR assay was found to be 10 copies (37attg), and the amplification plot of fluorescence signal versus cycle number showed the expected dose dependence curve (Fig. 3).

**Fig. 4:**
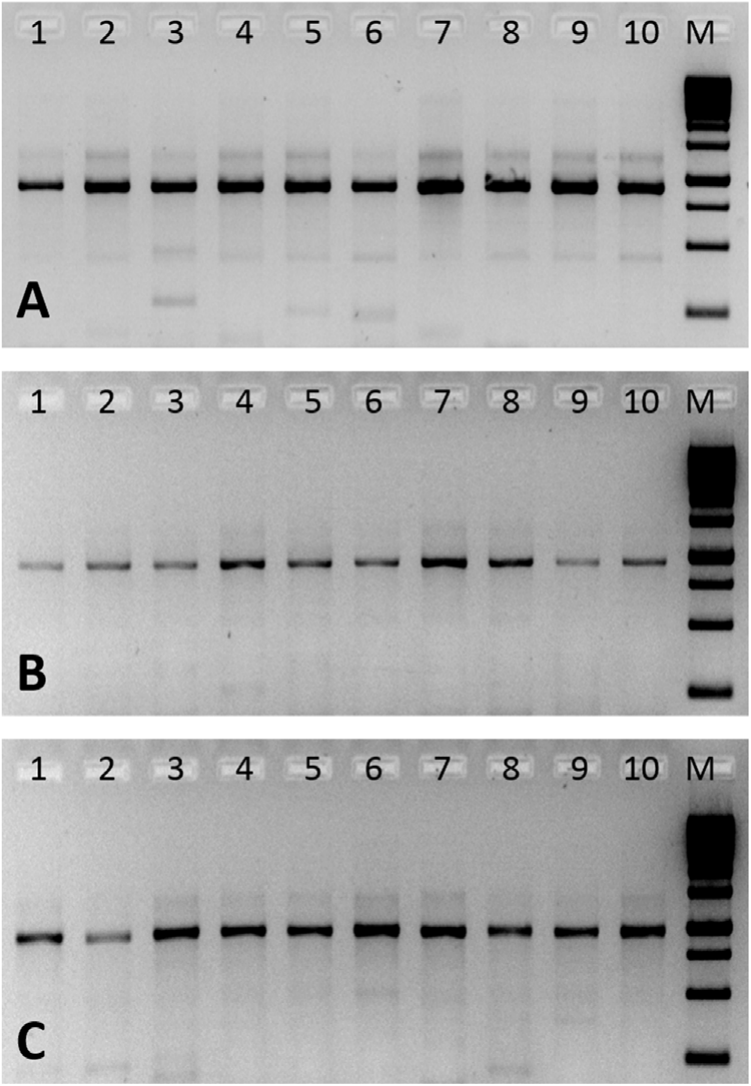
Agarose gel electrophoresis of HLA Class I genes PCR of gDNA samples from 10 HLA donors. PCR’s were done after normalization of the gDNA’s to 100 F13 copies (equivalent to 0.2 ng gDNA’s). Panel A: HLA-A PCR of all ten gDNA samples; Panel B: HLA-B PCR of all the ten gDNA samples; Panel C: HLA-C PCR of all the ten gDNA samples. M: DNA molecular weight marker (250 bp–10 Kb).

**Fig. 5:**
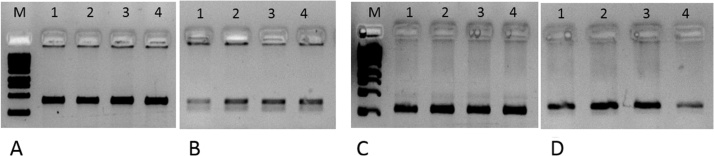
Agarose gel electrophoresis of HLA Class II genes PCR of gDNA samples from 4 HLA donors. PCR’s were done after normalization of the gDNA’s to 100 F13 copies (equivalent to 0.2 ng gDNA’s). Panel A: HLA-DPB1, exon2; panel B: HLA-DPB1, exon 3; panel C: HLA-DRB1, exon2 and panel D: HLA-DRB1, exon 3. Lanes 1–4 represent randomly chosen template gDNA’s from four donor samples; M: DNA molecular weight marker (250 bp–10 Kb).

All the 10 gDNA samples taken for this study showed variable gDNA concentrations when estimated by Nanodrop Spectrophotometry (Table 2). However, these samples showed significantly different F13 copy numbers, and the values did not correlate with the observed A260/280 ratios. When all these samples were normalized for 100 copies of F13 (equivalent to 0.2 ng of gDNA) and subjected to HLA PCR, it was observed that the PCR yielded signals of nearly equal intensity (Fig. 4). The PCR of exons 2 and 3 of DPB1 and DRB1 was also found to be similar intensity (Fig. 5), when they were normalized for 100 copies of F13.

**Table 2:** *F13A1* copy number determination for 10 gDNA samples by Q-PCR per 10 ng of genomic DNA.

S.N.	Sample details	Sample Type	Ct	Conc. gDNA^a^ ng/μl	*F13A1* copies^b^/10ng
1	Sample 12711	Test 1	25.27	55.7	6589
2	Sample 12712	Test 2	25.61	49.0	5410
3	Sample 12713	Test 3	25.34	44.9	6346
4	Sample 12714	Test 4	25.12	46.5	7203
5	Sample 12715	Test 5	25.19	48.2	6938
6	Sample 12716	Test 6	25.44	80.1	5987
7	Sample 12717	Test 7	25.28	49.2	6585
8	Sample 12718	Test 8	24.92	50.4	8108
9	Sample 12719	Test 9	25.07	61.8	7426
10	Sample 12720	Test 10	25.22	50.5	6806

a Genomic DNA concentration estimated by Nanodrop Spectrophotometry at 260 nm.

b The *F13A1* copy number in gDNA samples was determined using a standard curve prepared using pTZ57R/T-*F13A1* plasmid with known *F13A1* copy numbers ranging from 10^5^ to 10^1^ copies, in 10-fold dilutions. The *F13A1* copies in the standards were estimated using the formula: DNA (copies) = 6.022 × 10^23^ (copies mol^−1^) × DNA amount (g) DNA length (bp)  × 660 (g mol^−1^ bp^−1^).

## Discussion

The recent development of second-generation sequencing methods has brought in the possibility of sequencing a single DNA strand in isolation in contrast to the Sanger’s sequencing where such a possibility does not exist. For next-generation sequencing (NGS), the primary requirement is equimolar pooling of the amplicons for unambiguous HLA typing. From the HLA PCR optimization experiments carried in laboratory, it was clear that the PCR signals of various genes of HLA were not constant in spite of taking an equal concentration of gDNA for every target based on A260 nm readings. We observed that the signals varied from sample to sample, and the variations were not target gene dependent. We envisaged that the only factor that could contribute to such discrepancies would be unequal amounts of gDNA for every reaction. Since the gene locus for FXIIIa is highly polymorphic at the DNA level and is an extremely useful genetic marker on the short arm of chromosome 6 and the HLA genes also are localized on the same chromosome [11], we hypothesized that one could identify the copy number of F13 in the genomic samples and then F13-equivalent gDNA could be safely used for achieving similar PCR amplification of different HLA target genes. Moreover, there are advantages of using F13 as IC in our case since it does not require any reagents, gene products or plasmid to be added extraneously. This is because F13 can be easily amplified directly from the same source of gDNA samples that are intended for HLA typing by PCR.

The method of F13 Q-PCR described here does not form primer-dimer; hence, during PCR, one could expect formation of specific PCR product since primer-dimer competes with the target product and causes reduced amplification efficiency. Also, we observed clear and clean PCR signals of various HLA genes using merely 100 copies of the F13 equivalent gDNA preparations (0.2 ng of gDNA), which is five times less the amount of gDNA requirement than used by Ozaki et al. [12]. The report of effective and specific HLA PCR of Class I exons with merely 0.2 ng gDNA per reaction is hitherto unreported and is certainly cost-saving and encouraging.

HLA genes have a complex structure and have sequence homology among the different HLA class I loci, including the less polymorphic HLA-E, F, G genes and several pseudogenes [13]. It has been reported that the class I genes namely HLA-A, HLA-B, HLA-C and DRB1 affect the clinical outcomes of hematopoietic stem cell transplantation, and the class II genes like HLA-DQ and HLA-DP are not critical [14], we restricted the study with examining the F13 copy equivalents for PCR of exons 2 and 3 of HLA- Class I genes namely HLA-A, -B and –C and DPB1 and DRB1 of HLA Class II genes in this study.

FXIII A phenotype analysis in bone marrow transplant patients has identified monocytes/macrophages and/or megakaryocytes/platelets as a source of FXIII A1 along with liver as an extra hematopoietic source of FXIII A [15,16]. These data support the observed linkages of Factor XIII with HLA by Zhogbhi et al. [11], and hence, our work on the use of F13 as an internal control for HLA typing assumes critical importance. To the best of our knowledge, the use of F13 as an internal control is the first report and would be an ideal quantitative and economical method for using the right amount of gDNA from whole blood and mammalian cells for PCR-based diagnostics, forensic samples and other downstream applications.

Various kinds of ICs are known in the literature. Some of the housekeeping genes commonly used as expression controls in mammalian cells include glyceraldehyde-3-phosphate dehydrogenase (GAPDH), β-actin, β_2_-microglobulin, cyclooxygenase 1, hypoxanthine phosphoribosyl transferase 1, glucose-6-phosphate dehydrogenase, cyclophilin A, tubulin, transferrin receptor, and 18S ribosomal RNA [17]. Although the products of these genes are constitutively expressed, researchers have reported variations in the levels of their messenger RNA in certain clinical/experimental conditions that would affect the normalization [18]. Some workers have used internal control RNAs from coliphages as ICs [19] while Sachadyn and Kur [20] have used an IC where the primers possessed 5′ over-hanging ends identical to the primers used in the diagnostic reaction, whereas their 3′ ends were complementary to a predetermined DNA sequence of defined length and sequence. These are termed as internal amplification controls (IACs). However, the simultaneous amplification of two different DNA fragments flanked by the same primer sites results in either inhibition or enhancement of one or both products depending on the molar ratio of those DNA fragments. There is a single report on the use of human growth hormone (hGH) gene used as an IC [21], where the HLA products were differentiated from the hGH gene by melt curve analysis.

All the ICs described above have their own advantages and disadvantages. While looking for alternate candidates to be used as reference/housekeeping standards for our HLA PCR with a sole aim to resolve the issue of inconsistent PCR signals during HLA PCR, we came across a report of Khare et al. [10]. This report focuses on the use of short tandem repeat (STR) coagulation Factor XIII (F13) as a marker for gene amplification studies from blood and salivary DNA. This report intrigued us to explore the possibility of using F13 as an IC for our HLA PCR studies.

The human major histocompatibility complex is called the HLA (Human Leukocyte Antigen) maps to the short arm of chromosome 6 (6p21) and spans approximately 3600 kilobases of DNA [22]. The diversity of HLA polymorphism is predominant within the six classical HLA genes: the class I genes HLA-A,-B and-C and the class II genes HLA-DRB1,-DQB1 and -DPB1. The methods used to identify these regions for HLA typing include the sequence-based typing (SBT) technology followed by Sanger’s sequencing and the NGS.

To ensure successful amplification of all the input DNAs, Bowles and co-workers [23] used a 394 bp fragment of the Coxsackie-adenovirus receptor gene as an IC while β-actin and β-globin genes have been used as ICs by others [24]. Recently, Vinayagamoorthy et al. [25] described a method of using an IC where the analyte and the IC DNA shared the same PCR and sequencing annealing sequences. Our report on the successful use of F1 gene as an IC for such downstream application is new and applicable to work related to similar aspects.

## Conflict of interest

The authors declare no conflict of interest.

## Figures and Tables

**Figure:**