# Supplementary material for: Use of coagulation factor XIII (F13) gene as an internal control for normalization of genomic DNA’s for HLA typing
Source: MethodsX. 2018 Aug 3;5:881–9. doi: 10.1016/j.mex.2018.07.020 (PMC6107889; doi:10.1016/j.mex.2018.07.020)
Supplement: Supplementary file 1 [file mmc1.docx]

**Supplementary figure**

**Fig. S1 NCBI BLAST DNA sequence of the F13 PCR product.** Note that the homology of the F13 amplicon is 99% in comparison to the F13A1 sequence deposited in the NCBI database.
